# Adaptive and Specialised Transcriptional Responses to Xenobiotic Stress in *Caenorhabditis elegans* Are Regulated by Nuclear Hormone Receptors

**DOI:** 10.1371/journal.pone.0069956

**Published:** 2013-07-26

**Authors:** Laura M. Jones, Samantha J. Rayson, Anthony J. Flemming, Peter E. Urwin

**Affiliations:** 1 School of Biology, University of Leeds, Leeds, United Kingdom; 2 Syngenta, Jealott’s Hill International Research Centre, Bracknell, United Kingdom; University of Texas Health Science Center, United States of America

## Abstract

Characterisation of the pathways by which xenobiotics are metabolised and excreted in both target and non-target organisms is crucial for the rational design of effective and specific novel bioactive molecules. Consequently, we have investigated the induced responses of the model nematode *Caenorhabditis elegans* to a variety of xenobiotics which represent a range of putative modes of action. The majority of genes that were specifically induced in preliminary microarray analyses encoded enzymes from Phase I and II metabolism, including cytochrome P450s, short chain dehydrogenases, UDP-glucuronosyl transferases and glutathione transferases. Changes in gene expression were confirmed by quantitative PCR and GFP induction in reporter strains driven by promoters for transcription of twelve induced enzymes was investigated. The particular complement of metabolic genes induced was found to be highly contingent on the xenobiotic applied. The known regulators of responses to applied chemicals *ahr-1, hif-1, mdt-15* and *nhr-8* were not required for any of these inducible responses and *skn-1* regulated GFP expression from only two of the promoters. Reporter strains were used in conjunction with systematic RNAi screens to identify transcription factors which drive expression of these genes under xenobiotic exposure. These transcription factors appeared to regulate specific xenobiotic responses and have no reported phenotypes under standard conditions. Focussing on *nhr-176* we demonstrate the role of this transcription factor in mediating the resistance to thiabendazole.

## Introduction

All organisms regularly encounter exogenous compounds that must be metabolised and excreted. The general molecular mechanism of the metabolic response to such compounds is conserved between species and is divided into three successive phases. In Phase I, functional groups, often hydroxyl groups, are introduced into the xenobiotic compound. These groups are often required for entry into Phase II metabolism, which involves conjugation of these compounds to charged species such as glutathione and sugars for enhanced solubility. The major classes of enzymes involved in Phase I metabolism are cytochrome P450s (CYPs) and short-chain dehydrogenase/reductases, including alcohol dehydrogenases. Glutathione transferases (GSTs) and UDP-glucuronosyl transferases (UGTs) catalyse the conjugation reactions which occur in Phase II metabolism. The resulting soluble metabolites are then excreted by multi-drug efflux pumps, including ATP-binding cassette transporters during Phase III metabolism. Nuclear hormone receptors (NHRs) play a central role in regulation of the xenobiotic response in mammals; in particular the pregnane X receptor (PXR) and constitutive androstane receptor (CAR) family. Once bound to a xenobiotic, these receptors act as transcription factors and induce the expression of genes that encode metabolic enzymes and components of multi-drug efflux pumps (reviewed in [1]).

Whilst the genomes of human, mouse and *Drosophila* contain only 48, 49 and 18 predicted members respectively, *Caenorhabditis elegans* contains a massively expanded family of NHRs with 284 predicted members [2], [3]. Fifty of these NHRs have detectable phenotypes in RNAi screens or deletion mutants under standard conditions [4] and only 15–20 *C. elegans* NHRs are conserved among animal phyla [5], [6]. A comparable number of predicted NHRs is found in the genomes of other *Caenorhabditis* species but the NHR complement is highly reduced in the genomes of parasitic nematodes, with only 92, 76 and 27 predicted for *Meloidogyne incognita*, *M. hapla* and *Brugia malayi* respectively [7]. In *C. elegans,* DAF-12, NHR-48 and NHR-8 were originally identified as PXR/CAR homologues [3], [8]. DAF-12 is known to regulate dauer formation and is liganded by a sterol compound [9], [10] whilst NHR-48 appears to be involved in reproduction [11], [12]. NHR-8 has remained the only NHR with a known function in xenobiotic metabolism and is required for wild-type levels of resistance to chloroquine and colchicine [8]. In *Drosophila* the transcriptional response to phenobarbitol exposure relies, at least in part, on DHR96, which is an orthologue of the vertebrate PXR and CAR and the only NHR with a known role in detoxification in *Drosophila* [13]. In mammals the aryl hydrocarbon receptor (AHR), which is a member of the basic helix–loop–helix PER-ARNT-SIM (bHLH-PAS) superfamily also plays a role in xenobiotic metabolism [14]. Mammalian AHR directly binds a wide range of xenobiotics and regulates transcription of a distinct set of Phase I, II and III genes as well as displaying extensive cross-talk with CAR and PXR [15], [16]. AHR is also conserved in both *C. elegans* and *Drosophila* and, like the vertebrate form, it is expressed in chemosensory neurons [17], [18]. In *Drosophila* AHR partially mediates toxicity to benzene, toluene and xylene [19] but in *C. elegans* AHR is not associated with xenobiotic metabolism and has a role in controlling neuronal development [20]. Furthermore, *C. elegans* AHR-1 does not bind dioxins and related chemicals as vertebrate homologues do [21] and it is not activated by β-naphthoflavone in a yeast expression system [17] as found in vertebrates [22].

The response to different xenobiotics appears to be complex and specific in *C. elegans*. One wide-scale gene expression analysis showed that of 203 genes that responded to 48±5 hours exposure to β-naphthoflavone, fluoranthene, atrazine, clofibrate and diethylstilbestrol only 26 were induced by more than one chemical [23]. Differences in induction by a wide range of chemicals have also been found between CYP genes using RT-PCR [24] and Green Fluorescent Protein (GFP) reporter strains [25]. It seems unlikely that such a complex response is regulated by a single PXR/CAR-like regulator (NHR-8) and there are other classes of transcription factor that have been shown to regulate metabolic gene expression. The mediator subunit MDT-15 appears to regulate a number of genes encoding cellular metabolic enzymes (independently of NHR-8) including CYPs, UGTs and GSTs in response to fluoranthene but not β-naphthoflavone [26]. This coregulator also interacts with the sterol response element binding protein SBP-1 and NHR-49 during fatty acid metabolism [27], [28]. It is possible that MDT-15 may interact with NHRs during xenobiotic regulation since mammalian MED1/TRAP220 implements systemic detoxification through PXR and CAR [29], [30]. Other transcription factors may also regulate some xenobiotic responses. For example, a *C. elegans* homologue of NRF2 BZIP transcription factors in mammals, SKN-1, functions in the p38 MAPK pathway in parallel to the DAF-2-mediated insulin/IGF-1-like signalling pathway to regulate the oxidative stress response and longevity [31], [32]. SKN-1 has been shown to regulate GFP induction in reporter strains driven by *gst-4*, *gst-30* and *ugt-13* promoters under exposure to allyl isothiocyanate [33]. Together with the orthologue of the mammalian hypoxia-induced factor HIF-1 (a member of the bHLH-PAS superfamily) this transcription factor also regulates the response to hydrogen sulphide [34]. NRF2 is a central regulator of the oxidative stress response in mammals [35] and a NRF2 orthologue regulates the majority of transcriptional responses to phenobarbital, chlorpromazine and caffeine in *Drosophila* [36]. However, the ability of these other classes of transcription factor to bind xenobiotics directly in *C. elegans* – as NHRs can – is not clear and so they cannot necessarily explain specific responses to particular xenobiotics. For example, HIF-1 α-subunits may either directly sense oxygen or be influenced by an oxygen-sensing protein [37]. This, coupled to the massive expansion of NHRs and divergence in their lipid binding domains [38] encourages the speculation that, in *C. elegans* at least, other NHRs may be involved in orchestrating metabolism.

A number of wide-scale studies have been carried out to investigate transcriptional responses of *C. elegans* to a variety of different chemical exposures, including anthelmintics [39]–[41], insecticides [42], polychlorinated biphenyls [43], vertebrate steroids [44] and juglone [45]. Whilst these studies have identified a number of upregulated transcripts (particularly from the families involved in cellular metabolism) they have usually involved exposures to relatively few compounds. Furthermore only one exposure for each compound has generally been used, the time periods of which have also varied between studies. To uncover the complexity of metabolic induction in *C. elegans* we selected four unrelated, bioactive molecules to use as a ‘model system’ for multi-xenobiotic induction. These xenobiotics were the broad-spectrum pesticide dazomet and more specific pesticides; thiabendazole and imidacloprid, as well as the widely used chloroquine antimalarial. Preliminary microarray analyses had indicated that the majority of changes in expression were for those genes involved in Phase I and Phase II cellular metabolism (unpublished results) and these changes were confirmed by quantitative real-time PCR (qPCR). Twelve GFP reporter constructs driven by CYP, GST and UGT promoters were generated and used to investigate specific chemical induction of a selection of these genes. Four of the reporter strains were used in RNAi screens to uncover a number of transcription factors which could be regulating them in response to specific xenobiotic exposure. Phenotypes under combined xenobiotic exposure and RNAi were investigated for these transcription factors.

## Results

### Xenobiotic Induction of Transcripts Encoding Phase I and Phase II Metabolic Enzymes is Highly Specific

*C. elegans* were exposed to four xenobiotics and the effects on expression of genes involved in Phase I and Phase II cellular metabolism which had been upregulated ≥5-fold in preliminary microarray analyses were determined by qPCR. Two different exposures were used; one hour at the minimum xenobiotic concentration to have an observed effect on behaviour and 48 hours at the highest concentration at which nematodes retained some motility. Gene expression following xenobiotic exposure was compared to that in nematodes from control cultures exposed to 0.5% DMSO for the appropriate period. Chloroquine, dazomet and imidacloprid induced higher expression of genes after a 48 hr exposure than a 1 hr exposure at a lower concentration, whereas thiabendazole induced higher expression of genes after the shorter, weaker exposure (Table S1). Interestingly imidacloprid significantly induced the expression of nine CYP genes, whilst thiabendazole, chloroquine and dazomet induced the expression of only four, three and one CYP genes respectively (p≤0.01). Conversely dazomet induced the expression of more genes belonging to Phase II cellular metabolism than chloroquine, imidacloprid and thiabendazole. Dazomet induced expression of 11 GSTs and two UGTs whilst imidacloprid and thiabendazole induced the expression of four and two UGTs respectively without any GST induction (p≤0.01). Chloroquine did not induce expression of any UGTs or GSTs. Furthermore, the induction of individual genes was highly specific to the applied xenobiotic, particularly after the shorter exposure. Of the 33 genes with increased expression under xenobiotic exposure only four of these were induced (p≤0.01) by more than one chemical (*cyp-35a3*, *-a5*, -*b2* and *ugt-13*).

**Figure 1:**
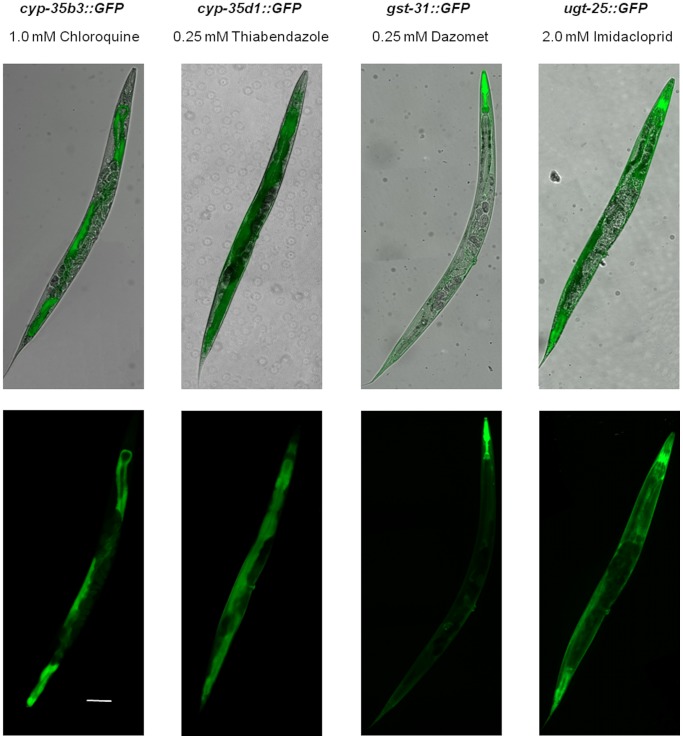
Expression of GFP in a selection of reporter strains driven by promoters for genes encoding CYP, GST and UGTs. Images with UV and visible illumination of *cyp-35b3::GFP*, *cyp-35d1::GFP*, *gst-31::GFP* and *ugt-25::GFP* reporter strains in adult stage under exposure to 1.0 mM chloroquine, 0.25 mM thiabendazole, 0.5 mM dazomet and 2.0 mM imidacloprid, respectively. An example of each of the spatial expression patterns observed is shown; intestine (*cyp-35b3::GFP* and *cyp-35d1::GFP*) pharynx/hypodermis/vulva (*gst-31::GFP*) and hypodermis/vulva (*ugt-25::GFP*). Scale bar represents 100 µm in all images.

**Figure 2:**
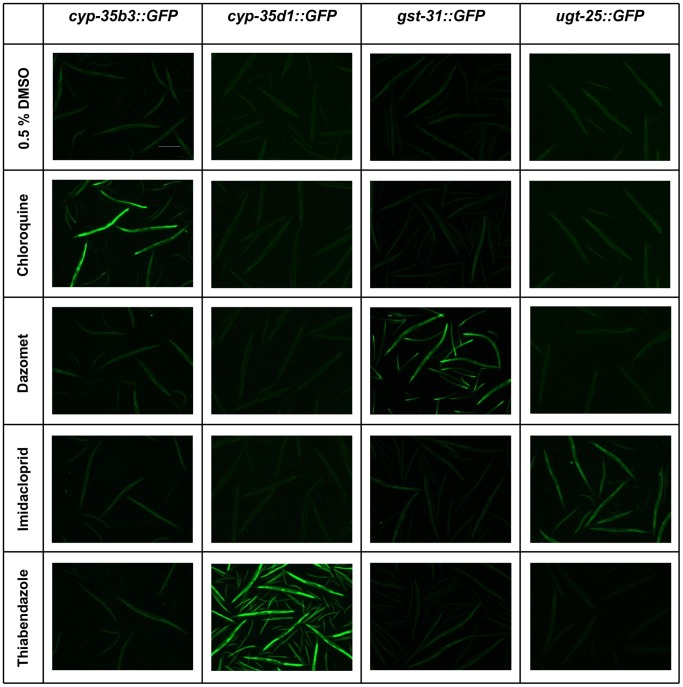
The response of GFP reporter strains under xenobiotic exposure. Low magnification images with UV illumination of *cyp-35b3::GFP*, *cyp-35d1::GFP*, *gst-31::GFP* and *ugt-25::GFP* reporter strains in adult stage under exposure to 0.25 mM beta-napthoflavone, 1.0 mM chloroquine, 0.5 mM dazomet, 0.25 mM juglone, 2.0 mM imidacloprid and 0.25 mM thiabendazole. Scale bar represents 500 µm in all images.

The specificity of the xenobiotic response was confirmed for 12 metabolic genes following generation of *C. elegans* reporter strains in which *gfp* expression was driven by promoters for *cyp-34a7*, *cyp-35a5*, *cyp-35b1*, *cyp-35b3*, *cyp-35d1*, *ugt-8*, *ugt-13*, *ugt-25*, *ugt-37*, *gst-25, gst-30* and *gst-31*. There was limited background expression of GFP in any of the strains, however strong expression was observed in the intestine, pharynx, vulva and/or hypodermis when the transgenic strains were exposed to specific xenobiotics (Figure 1). GFP expression was strongly inducible in *cyp-35a5::GFP* and *cyp-35d1::GFP* by only thiabendazole, in *cyp-35b1::GFP* and *cyp-35b3::GFP* by chloroquine, in *cyp-34a7::GFP*, *ugt-25::GFP* and *ugt-37::GFP* by imidacloprid and in *gst-25::GFP*, *gst-30::GFP* and *gst-31::GFP* by dazomet (Table 1 and Figure 2). Both thiabendazole and imidacloprid induced GFP expression in *ugt-8::GFP* whilst thiabendazole and dazomet induced expression of *ugt-13::GFP* (Table 1 and Figure 2). The specific responses of each reporter strain therefore correlated with the upregulation of gene expression determined by qPCR analysis.

**Table 1:** GFP expression in reporter strains driven by promoters for genes significantly upregulated (p≤0.01) under xenobiotic exposure in qPCR analyses.

Promoter	Chemical exposure										Location
	DMSO control		Chloroquine		Dazomet		Imidacloprid		Thiabendazole		
	1 hr	48 hr	1 hr	48 hr	1 hr	48 hr	1 hr	48 hr	1 hr	48 hr	
*cyp-34a7*	1	1	1	1	1	1	1	3	1	1	Intestine
*cyp-35a5*	1	1	1	1	1	1	1	2	5	3	Intestine
*cyp-35b1*	1	1	1	5	1	1	1	2	1	1	Intestine
*cyp-35b3*	0	0	1	5	0	0	0	1	0	0	Intestine
*cyp-35d1*	0	0	0	0	0	0	0	2	5	4	Hypodermis, Intestine, Vulva
*ugt-8*	1	1	1	1	1	1	1	2	3	1	Intestine, Pharynx
*ugt-13*	1	1	1	1	3	4	1	1	2	1	Hypodermis, Tail, Vulva
*ugt-25*	1	1	1	1	1	1	1	4	1	1	Hypodermis, Tail, Vulva
*ugt-37*	0	0	0	0	0	0	0	2	0	0	Pharynx, Tail, Vulva
*gst-25*	0	0	0	0	1	3	0	0	0	0	Hypodermis, Pharynx, Tail, Vulva
*gst-30*	0	0	0	0	1	3	0	0	0	0	Pharynx, Tail, Vulva
*gst-31*	0	0	0	0	1	5	0	0	0	0	Hypodermis, Pharynx, Tail, Vulva

(0 = no expression, 5 = strong expression).

### Inducible Metabolic Responses Reported here are not Dependent on AHR-1, HIF-1, MDT-15 and NHR-8

**Figure 3:**
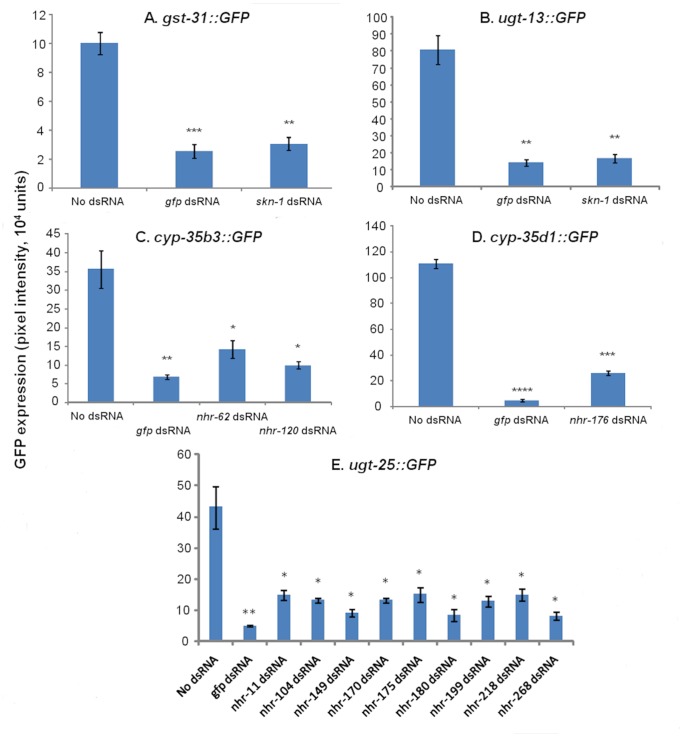
GFP expression upon xenobiotic induction in reporter strains driven by promoters for A) *gst-31* B) *ugt-13* C) *cyp-35b3* D) *cyp-35d1* E) *ugt-25* following exposure to dsRNA to knockdown transcription factors. Xenobiotic exposures were A) and B) 0.5 mM dazomet, C) 1.0 mM chloroquine, D) 0.25 mM thiabendazole and E) 2.0 mM imidacloprid. The mean of three biological replicates is plotted with standard error of the mean. *p<0.05, **p<0.01 and ***p<0.001 from one-way Analysis of Variance.

Predicted SKN-1 binding sites above a threshold of 85.0 were identified in the promoter regions of *cyp-35a5* (2 sites), *cyp-35b1* (1), *cyp-35b3* (4), *cyp-35d1* (3), *gst-25* (3), *gst-30* (5), *gst-31* (3), *ugt-8* (1), *ugt-13* (4) and *ugt-25* (5). However, when *skn-1* was knocked down in each reporter strain by RNAi (76±5% reduction in transcript) prior to induction by dazomet, GFP expression was only reduced in *gst-31::GFP* and *ugt-13::GFP* (as previously observed [33], [46]) (Figure 3). To determine whether or not any xenobiotic inductions are dependent on *ahr-1, hif-1*, *mdt-15* or *nhr-8* these transcription factors were also knocked down by RNAi in the transgenic reporter strains but GFP expression in all strains remained inducible (results not shown). Efficiency of knockdown was confirmed for *mdt-15* and *nhr-8* by observation of phenotypes and qPCR confirmed knockdown for *ahr-1* and *hif-1* (81±4 and 65±5% respectively).

### RNAi Screening has Identified Transcription Factors Regulating Specific Chemical Induction of GFP Reporters Driven by Promoters for Cellular Metabolic Enzymes

**Figure 4:**
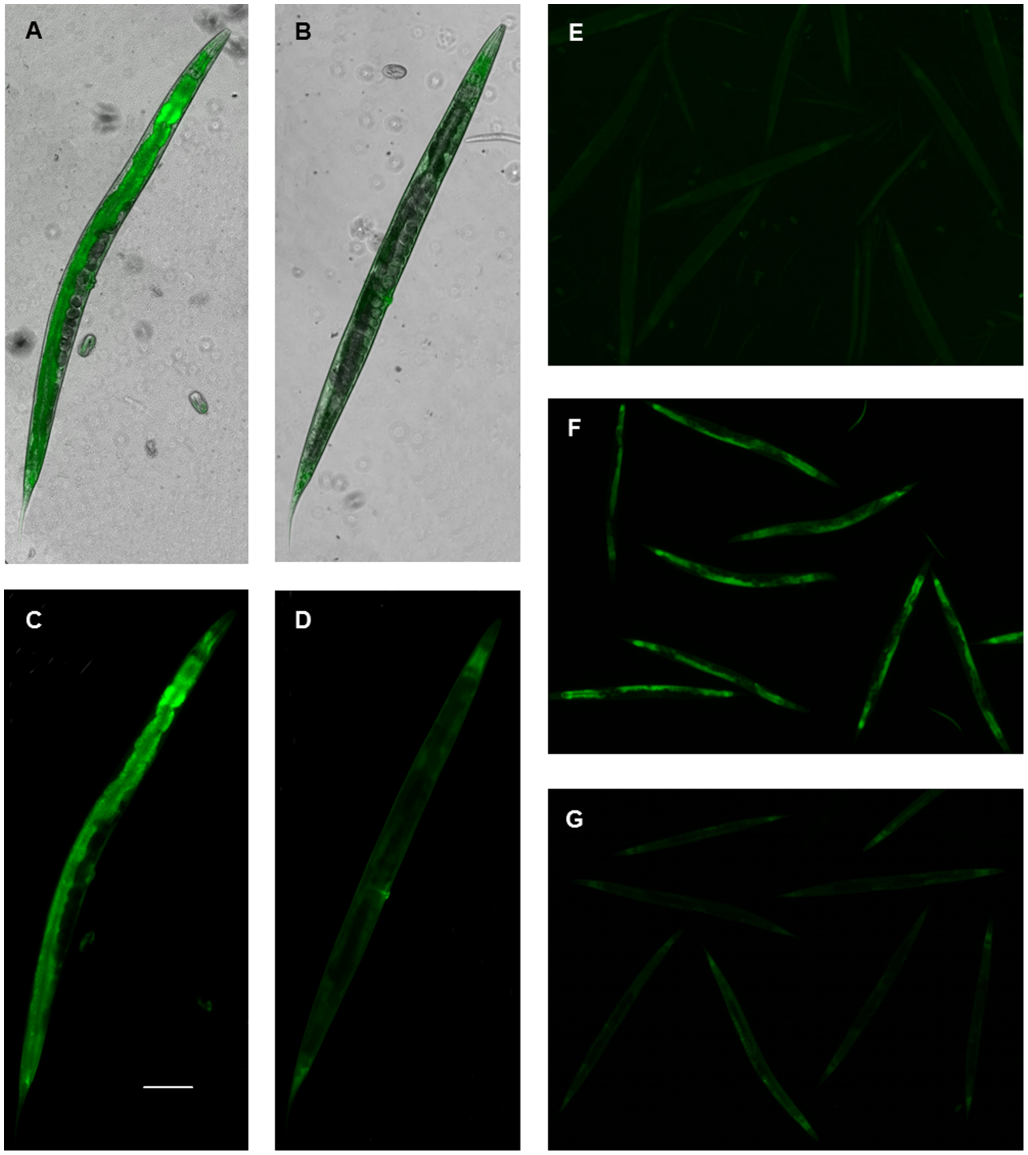
GFP expression of reporter lines when NHR-176 was knocked down by RNAi prior to chemical induction. GFP expression in *cyp-35d1::GFP* adult stage upon induction by 0.25 mM thiabendazole when fed on *E. coli* HT115 (DE3) containing *pl4440* (control; A and C) and when fed on *E. coli* HT115 (DE3) containing *pl4440::nhr-176* (B and D). Low magnification images showing GFP expression in *cyp-35d1::GFP* adult stage under UV illumination (E) upon induction by 0.5% DMSO when fed on *E. coli* HT115 (DE3) containing *pl4440* (F) upon induction by 0.25 mM thiabendazole when fed on *E. coli* HT115 (DE3) containing *pl4440* (G) upon induction by 0.25 mM thiabendazole when fed on *E. coli* HT115 (DE3) containing *pl4440::nhr-176* (F). Scale bar represents 100 µm in A-D.

Reporter strains induced most strongly and specifically by xenobiotic exposure (*cyp-35b3::GFP*, *cyp-35d1::GFP*, *gst-31::GFP* and *ugt-25::GFP*) were selected for RNAi screens to target transcription factors under chloroquine, thiabendazole, dazomet and imidacloprid exposure respectively. Wide-scale RNAi screens targeting 387 transcription factors identified 12 which could be regulating these promoters under chemical exposure. Predicted sites for binding of CdxA, GATA and HSF transcription factors were identified in the promoter regions of *cyp-35b3* (4 sites), *cyp-35d1* (3) and *ugt-25* (5) yet none of these regulated any chemical inductions of reporters driven by these promoters (results not shown). Nine transcription factors were implicated in the response to imidacloprid in *ugt-25::GFP*, two for the response to chloroquine in *cyp-35b3::GFP*, one was required for the response to thiabendazole in *cyp-35d1::GFP* and one for the response to dazomet in *gst-31::GFP* and *ugt-13::GFP* (see Figure 3). RNAi knockdown of the transcription factors required for the responses to chloroquine, imidacloprid and thiabendazole resulted in a similar level of reduction in GFP expression in each case (68±8%, p<0.05) although this was not as high as the reduction achieved following direct RNAi knockdown of *gfp* (89±9%, p<0.01). Similar levels of reduction in GFP expression were found in *gst-31::GFP* and *ugt-13::GFP* following RNAi knockdown of *skn-1* (72±5%, p<0.01 and 79±3%, p<0.01) and *gfp* (81±2%, p<0.01 and 75±5%, p<0.01). Apart from *skn-1* all of these transcription factor genes encode nuclear hormone receptors and have no detectable phenotypes in mutants or when knocked down by RNAi under standard conditions. GFP expression in the intestine was highly reduced when *nhr-176* was knocked down in *cyp-35d1::GFP* prior to induction by thiabendazole (Figure 4) which supports the previous finding that this transcription factor is enriched in the intestine [47]. Furthermore, no transcription factors were found that appeared to regulate more than one of the four chemical responses. Interestingly knock down of the NHRs which resulted in reduced GFP induction in *cyp-35b3::GFP*, *cyp-35d1::GFP* and *ugt-25::GFP* did not result in reduced GFP induction in *cyp-34a7::GFP*, *cyp-35a5::GFP*, *cyp-35b1::GFP*, *ugt-8::GFP* and *ugt-37::GFP* under the same chemical exposure. BLAST searches of translated genome sequences for *C. briggsae* and *C. remanei* with translated mRNA sequences from *C. elegans* indicated that eight of these twelve NHRs have direct homologues in both *Caenorhabditis* species and a further two NHRs have direct homologues in one *Caenorhabditis* species (Table S2).

### RNAi Knockdown of *nhr-176* Results in Enhanced Susceptibility to Thiabendazole but not to 5-hydroxythiabendazole

**Figure 5:**
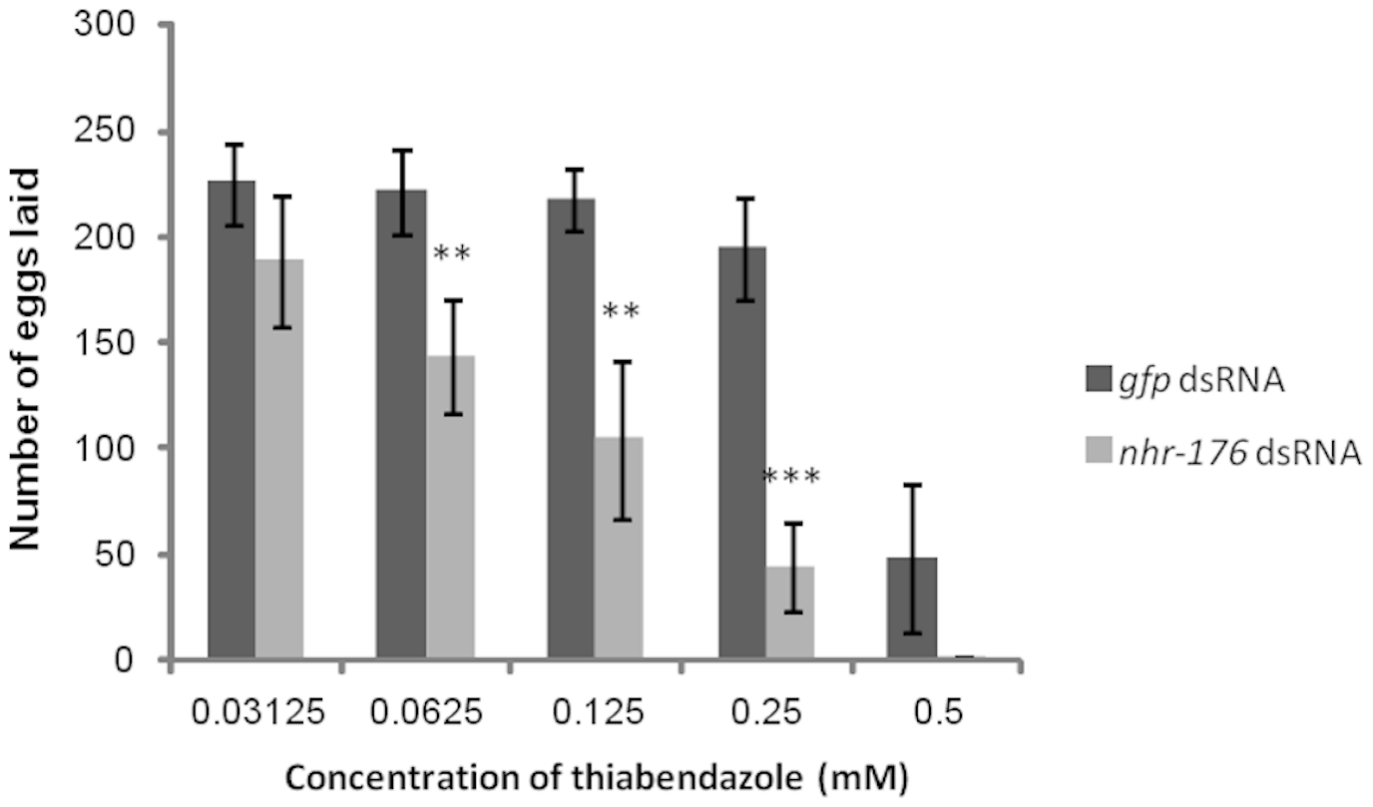
Total number of eggs laid by nematodes grown under RNAi and thiabendazole exposure from L1 stage for four days. The mean of three biological replicates is plotted with standard error of the mean. *p<0.05, **p<0.01 and ***p<0.001 from one-way Analysis of Variance.

To uncover the role of the 12 identified transcription factors in mediating whole-organism effects of xenobiotic exposure the effects on survival and reproduction were assessed after four days of exposure to both dsRNA and xenobiotic. Very few phenotypes under chemical exposure were detected when these twelve NHRs were knocked down individually by RNAi. However, knock down of the only regulator (*nhr-176*) found to be required for GFP expression in the *cyp-35d1::GFP* reporter strain rendered nematodes more susceptible than the controls to thiabendazole. Significantly fewer eggs were laid by nematodes exposed to *nhr-176* dsRNA and thiabendazole (≥0.062 mM) for four days from L1 stage (p≤0.01, Figure 5). Knockdown of *nhr-176* in the absence of xenobiotic did not have an effect on egg-laying. Interestingly, RNAi targeting of *nhr-176* did not alter the susceptibility of nematodes to 5-hydroxythiabendazole (data not shown); the main metabolite of thiabendazole in mammals [48].

## Discussion

This study is a first step in addressing how complex regulation of metabolism allows highly specialised xenobiotic responses in the nematode *C. elegans*. Different chemicals induced expression of different metabolic genes, with only four of the tested genes induced by more than one xenobiotic (*cyp-35a3*, *-a5*, *-b2* and *ugt-13*). Thiabendazole induced stronger expression of genes after a 1 hr exposure whereas chloroquine, dazomet and imidacloprid induced stronger expression of more genes after a 48 hr exposure (Table S1). This may suggest that thiabendazole is more rapidly metabolised than the other three xenobiotics. Exposure to thiabendazole, imidacloprid and chloroquine significantly induced transcription of several CYPs and UGTs but not any GSTs (Table 1 and Table S1). A similar induction profile has been found following a 4 hr exposure to albendazole, although the individual genes upregulated are not all the same as those induced by the closely related thiabendazole [39]. Conversely, dazomet exposure significantly induced transcription of eleven GSTs and two UGTs but only one gene from Phase I metabolism was significantly induced (Table S1). A previous study of wide-scale gene expression in response to 1 hr exposure to the oxidative stress inducer, juglone, also showed significant upregulation of transcripts for several GSTs identified in this study [45]. Increased GST expression has previously been observed following paraquat treatment in rodents [49]–[51] and in *Drosophila* [52]. Our data may indicate that dazomet is a substrate for Phase II metabolism in the absence of prior Phase I metabolism, explaining the induction of Phase II but not Phase I enzymes by this compound.

The majority of CYP isoforms which were strongly inducible by chloroquine, imidacloprid and thiabendazole belong to the CYP-35 subfamily. The three main isoforms induced under chloroquine exposure all belong to the CYP-35B subfamily (CYP-35B1, -B2 and –B3; Table 1). Other CYP isoforms appear to be responsible for thiabendazole metabolism, with transcript for CYP-35A5, - C1 and -D1 being the most strongly induced in *C. elegans* (Table S1 and Figure S4). The main metabolite of thiabendazole in mammals is 5-hydroxythiabendazole [48] and CYP-35A5, - C1 and -D1 may be involved in production of the hydroxylated form. The two UGTs (UGT-8 and UGT-13) induced by thiabendazole in *C. elegans* may catalyse production of a glucosylated form identified during albendazole metabolism in *C. elegans* [39]. The responses to these xenobiotics are yet to be investigated in *Drosophila* but homologues to the CYP3 subfamily in mammals have been associated with resistance to other xenobiotics including imidacloprid [53]. In mammals, the metabolism of imidacloprid is far more complex than that of chloroquine and thiabendazole, with many more metabolites having been identified. This may explain the induction by imidacloprid, particularly after 48 hrs, of several CYP and UGT members across different subfamilies in *C. elegans* during this study.

Members of CYP-2 and CYP-3 families in mammals are regulated by PXR and CAR during induction by a wide range of xenobiotics, whilst CYP-1 members are regulated by AHR [15]. However, RNAi knockdown of *nhr-8* was not required for the chemical induction of any reporter strains driven by CYP promoters or others; in this regard *C. elegans* appears different from mammals. NHR-8 does regulate a wide range of endogenous responses in *C. elegans* including fat metabolism [54]. The PXR/CAR homologue in *Drosophila*, DHR96, binds endogenous ligands including cholesterol [55] and regulates only ∼10% of the genes altered in response to phenobarbital [13]. Furthermore, knock-down of the mediator subunit *mdt-15* (65±5% transcript suppression) and the mammalian hypoxia-induced factor *hif-1* orthologue (65±6% transcript suppression), which have also been shown to regulate xenobiotic responses in *C. elegans* [26], [34] did not affect chemical induction in any of the reporter strains. NRF2 is a central regulator of the oxidative stress response in mammals [35] and its orthologue in *Drosophila* appears to regulate the majority of xenobiotic responses [36]. Although the knockdown of *skn-1* (by 76±5%) resulted in reduced chemical induction in reporters driven by *gst-31* and *ugt-13* promoters in both this study (Figure 3) and in a previous study [46] ^,^ [33] this nrf-2 homologue was not involved in any other chemical responses (data not shown), despite the occurrence of predicted SKN-1 binding sites in ten of the promoters used in reporter constructs. This study includes the first wide-scale RNAi screen to investigate the regulation of specific chemical induction in a selection of GFP reporter strains. It indicated that a number of other transcription factors (to which no function has been previously assigned) are involved in the regulation of xenobiotic responses. This is in contrast to *skn-1* which shows a strong phenotype under standard conditions when knocked down by RNAi [56] and in deletion alleles [57]. The regulation of GFP expression in reporter lines was highly specific, with no transcription factors involved in the response to more than one of the four chemicals; chloroquine, dazomet, imidacloprid and thiabendazole. Taken together our data imply a multi-regulator system controlling the induction of metabolic genes.

More transcription factors appeared to be regulating the response to chloroquine and imidacloprid, compared to thiabendazole and dazomet, where only one transcription factor was found for each. This may suggest that the responses to thiabendazole and oxidative stress involve fewer and/or less redundant transcription factors than those responses to chloroquine and imidacloprid. We describe, for the first time, the regulation of an anthelmintic response by a single NHR. Knockdown of *nhr-176* significantly enhanced susceptibility of *C. elegans* to thiabendazole (p<0.01, Figure 5). Since knocking down of *nhr-176* did not alter the susceptibility of nematodes to 5-hydroxythiabendazole this implies that *cyp-35d1* encodes an enzyme which catalyses the conversion of thiabendazole to its hydroxylated form. During knockdown of CYP-35D1 it is possible that CYP-35A5 catalyses some hydroxylation of thiabendazole since the reporter strain driven by the promoter for this gene remains inducible by thiabendazole under RNAi knockdown of *nhr-176*. Thiabendazole directly binds tubulin in *Haemonchus contortus* [58] and mutations in *ben-1* and *tub-1* confer resistance to thiabendazole in *C. elegans* [59] and *H. contortus* [60] respectively. Therefore thiabendazole itself may contribute to more toxicity in nematodes than oxidative damage and other effects from metabolised forms but this remains to be tested. The presence of a homologue for *nhr-176* in the closely related *C. briggsae* (as well as *C. remanei*) may explain the similar effect of thiabendazole on population growth in this species compared to *C. elegans* [61]. Direct homologues were found in both *C. briggsae* and *C. remanei* for eight of the twelve NHRs regulating xenobiotic metabolism in *C. elegans* and homologues for a further two NHRs were found in only one of the *Caenorhabditis* species (Table S2). This is more than the proportion of conservation found for the full complement of NHRs in *C. elegans*, where only half are conserved in both *C. briggsae* and *C. remanei* and a further 10% are conserved in only one of these *Caenorhabditis* species [62].

The lack of reduced GFP expression in other reporter strains inducible by chloroquine and imidacloprid when NHRs regulating the response to the same xenobiotic were targeted by RNAi could suggest that these downstream genes may catalyse different metabolic pathways of the same xenobiotic. For example, chloroquine induces both *cyp-35b1* and *cyp-35b3* but while *nhr-62* and *nhr-120* are required for the induction of *cyp-35b3*, they are not required for that of *cyp-35b1*. Similarly, NHRs required for the induction of *ugt-25* by imidacloprid are not required for the induction of *cyp-34a7* or *ugt-37* by the same compound. However, phenotypes in xenobiotic susceptibility were not detected for these transcription factors when knocked down by RNAi and a combination of transcription factors together may be required for a functional response. Alternatively, the involvement of multiple regulators may imply compensatory and/or functionally redundant regulatory pathways. It is also possible that other transcription factors may be required which were not represented in these RNAi screens. This may explain why RNAi knockdown of *nhr-176* reduced GFP expression less (∼77%, p<0.001, Figure 3C) than RNAi knockdown of *gfp* (∼96%, p<0.0001, Figure 3C) in *cyp-35d1::GFP* despite being the only transcription factor found regulating GFP induction in this reporter. It is also possible that some inductions are genuinely without function since *C. elegans* metabolism has not evolved in the presence of synthetic compounds and so might respond inappropriately. All of the NHRs identified as regulating one of these xenobiotic responses are members of the supplementary group of NHRs in nematodes which have evolved independently from an HNF4 ancestor through adaptive expansion [7]. In contrast to NHR-8 in *C. elegans*, PXR and CAR have been shown to regulate a wide range of xenobiotic responses in mice and rats [63], [64]. Although one PXR/CAR orthologue is associated with some xenobiotic metabolism in *Drosophila* [13] an NRF2 orthologue appears to regulate the majority of xenobiotic responses in this organism [36]. The xenobiotic response in free-living nematodes may be more specialised and involve more regulators than that in mammals and insects. *C. elegans* has evolved numerous NHRs which are not found in other metazoans and are poorly conserved even within the nematode phylum. We have shown that several of these are required for the induction of metabolic gene expression by xenobiotics. The adaptive significance of the use of additional NHRs in *C. elegans* and why it might have evolved remains unclear. It may be that they enable unique metabolic capabilities in *C. elegans*; comparing metabolite production in *C. elegans* and other metazoans would likely reveal this. Or they may allow an increased specificity of response to particular xenobiotics where the responses of other organisms are more generalised. Further understanding of these possibilities will enhance the utility of *C. elegans* as a model for metabolism and, furthermore, shed light on the evolution of metazoan metabolism.

### Conclusions

The xenobiotic response in *C. elegans* is highly specialised, contingent on the applied xenobiotic and controlled by multiple regulators. We show that regulators identified in previous studies of *C. elegans* (*ahr-1*, *hif-1, mdt-15* and *nhr-8*) are neither sufficient nor necessary to explain all the patterns of induction we see in GFP reporter strains. Furthermore, we identify twelve additional regulators of metabolic induction, the majority conserved in other *Caenorhabditis* species whilst others are unique to *C. elegans*. Finally, we describe for the first time, a transcription factor that modulates the susceptibility of *C. elegans* to an anthelmintic.

## Materials and Methods

### qPCR Analyses

Mixed stages of *C. elegans* wild-type strain N2 (Bristol) were cultivated in liquid S Basal medium (0.1 M NaCl, 0.05 M potassium phosphate pH6, 5 µgml^−1^ cholesterol (from a 5 mgml^−1^ stock in ethanol)) supplemented with 50 µgml^−1^ Nystatin (Sigma-Aldrich, UK) and 50 µgml^−1^ streptomycin (Sigma-Aldrich, UK) on a diet of *E. coli* HB101 as previously described [65]. Synchronised cultures were obtained by treatment of mixed-stage cultures with sodium hypochlorite and eggs were allowed to hatch overnight at 20°C in S Basal medium [66]. Approximately 200,000 L1 larvae were added to triplicate 500 ml cultures and incubated at 20°C in an orbital incubator (Beckman Ltd) operating at 200 rpm for 72 hrs. Each triplicate culture was divided between 10×50 ml aliquots in 250 ml flasks prior to xenobiotic exposure. Conditions used for xenobiotic exposures were one hour at the minimum concentration for the xenobiotic to have an observed effect on behaviour of ∼50% nematodes and 48 hours at the highest concentration at which ∼ 50% nematodes retained some motility when stimulated with a worm pick [67]; untreated control (0.5% DMSO, 1 and 48 hours), chloroquine (0.25 mM, 1 hour or 1 mM, 48 hours), dazomet (0.25 mM, 1 hour or 0.5 mM, 48 hours), imidacloprid (0.5 mM, 1 hour or 2 mM, 48 hours) and thiabendazole (0.125 mM, 1 hour or 0.25 mM, 48 hours). Nematodes were chilled at 4°C for 30 minutes, collected by centrifugation at 1200 *g* and separated from *E. coli* and debris by sucrose flotation [68]. Following one wash with chilled S basal nematodes were flash frozen in liquid nitrogen. Total RNA was extracted using an RNeasy kit (Qiagen). RNA integrity was assessed using a 2100 Bioanalyser (Agilent, UK) and cDNA was prepared using Superscript II Reverse Transcriptase (Invitrogen, UK). Gene expression of metabolic enzymes was assessed relative to the DMSO control and two stable transcripts (*ama-1* and *Y35g12.2*) were used as reference genes. The oligonucleotide sequences of all the primers are provided in Text S1. Brilliant III Ultra-Fast SYBR® Q-PCR Master Mix (Agilent, UK) was used without additional magnesium. The Bio-Rad CFX96 (Bio-Rad, UK) was programmed as follows; 3 minutes at 95°C followed by 40 cycles of 5 seconds at 95°C and 10 seconds at 60°C. Transcript expression was analysed using Bio-Rad CFX Manager 3.0 software.

### Generation of Promoter::Reporter Constructs

Promoterome clones and promoter::*gfp* fusions were generated as previously described [69]. Promoterome clones consisted of Multisite Gateway Entry Vector pDONR P4-P1R containing promoter regions upstream of *C. elegans* ORFs (http://worfdb.dfci.harvard.edu/promoteromedb/). Promoter sequences are provided in Text S2. LR clonase (Invitrogen) was used to transfer promoters from the Promoterome Entry clone to the Multisite Destination vector pDEST-DD04 (kindly provided by Prof. Ian Hope). DNA sequencing was performed (GATC Biotech, UK) to confirm Promoterome inserts prior to *C. elegans* transformation. Microprojectile bombardments were performed using the Bio-Rad PDS-1000/He with Hepta adapter as previously described [69]. Two 50 ml cultures of *C. elegans* strain DP38 (unc-119 (ed3) were grown in S Basal medium with shaking, at 20°C for seven days. Cultures were then transferred into 50 ml polypropylene tubes for adult and L4 stage nematodes to settle out, at room temperature, under gravity over 10 minutes. Approximately 7 µg of each plasmid was linearised by digestion with NgoMIV, HindIII or BamHI restriction enzyme in NEB buffer in a total reaction volume of 35 µl prior to precipitation onto gold particles for bombardment. 60 mg gold particles (0.3–3 µm; Chempur, Germany) were washed in 70% ethanol and resuspended in 1 ml sterile 50% glycerol. 30 µl of plasmid digest was added directly to 70 µl of gold bead suspension and vortexed for 1 minute. 300 µl of 2.5 M CaCl_2_ was added drop wise while vortexing to prevent sedimentation of the particles and 112 µl spermidine was added in the same way. After vortexing for 5 minutes the suspension was centrifuged at 2400 *g* for 5 seconds and the supernatant discarded. The gold beads were washed in 800 µl 70% ethanol, resuspended in 70 µl 100% ethanol and vortexed until use. A 9 cm NGM agar plate seeded with *E. coli* HB101 was inoculated with seven 150 µl aliquots of nematodes, placed in positions representing targets of the Hepta adapter. The bombardment procedure was then followed according to the manufacturer’s instructions. The inoculated NGM agar plate was placed on the second target shelf up in the Bio-RadPDS-1000/He and 9.3 MPa (1350 psi) rupture disks were used with a vacuum of 91 kPa (26 in. Hg). Following bombardment, 1 ml of M9 buffer (20 mM KH_2_PO_4_, 20 mM NaHPO_4_, 0.1 M NaCl and 1 mM MgSO_4_) was added to each plate and the nematodes were left to recover for 1 hour at 20°C. Nematodes were then washed from the plates with 4 ml M9 buffer and 0.5 ml of the nematode suspension was used to inoculate each of seven seeded 9 cm NGM plates. All eight plates, including the plate used in the bombardment, were incubated at 20°C for three weeks. Plates were then assessed for the presence of nematodes rescued for the *unc-119* mutant phenotype and displaying the wild-type phenotype. Four individuals from each large plate were transferred individually to seeded 5 cm NGM agar plates. After seven days the established lines were assessed for level of transmission of the rescued phenotype and up to eight independent lines were generated per bombardment. The *gst-30::GFP* strain was provided by Prof. David Baillie.

### Analysis of GFP Expression and RNAi

Nematodes displaying the wildtype phenotype were transferred to 96-well plates containing 100 µl M9 buffer with each xenobiotic at the same concentrations and time points used for qPCR analyses. GFP expression for each line was assessed using an Olympus SZX12 stereo-binocular fluorescent microscope and the most responsive lines were scored on an arbitrary scale from 0 (showing no GFP expression) to 5 (showing strong GFP expression). Images were captured through a Leica LEITZ DM RB microscope and GFP expression was analysed using Image-Pro Plus software (Media Cybernetics, USA). The RNAi transcription factor set containing 387 *E. coli* HT115 (DE3) clones was obtained commercially from Source Bioscience, UK (http://www.lifesciences.sourcebioscience.com/). Knock-down by RNAi was achieved by feeding as previously described [70]. *E. coli* HT115 (DE3) clones were grown in 96-well plates for eight hours in LB liquid containing 50 µgml^−1^ ampicillin. 5 µl of each culture was then seeded directly into 96-well plates containing NGM with 1 mM IPTG and 50 µgml^−1^ ampicillin prior to growing overnight at 25°C. Approximately ten arrested L1 stage *C. elegans* (from mixed stage cultures treated with sodium hypochlorite as previously described [66]) for each reporter strain were transferred to individual wells. Following four days of incubation at 20°C the resulting adults and young larvae were washed from each well in M9 buffer containing the appropriate xenobiotic and transferred to new wells. The concentrations of xenobiotic used for induction were 1 mM chloroquine, 0.5 mM dazomet, 2 mM imidacloprid and 0.25 mM thiabendazole. Each xenobiotic was dissolved in DMSO present at a final concentration of 0.5% and control cultures contained 0.5% DMSO only. Following a 24 hour exposure GFP expression in each well culture was assessed as previously described. Each RNAi screen was repeated at least three times. For assessment of phenotypes under xenobiotic exposure RNAi was carried out in the same way as described but xenobiotics were added directly to NGM in 48-well plates. Approximately 15 arrested L1 stage *C. elegans* wild-type strain N2 (Bristol) from hypochlorite treated cultures [66] were transferred to individual wells and the total number of progeny in each well was assessed after four days. At least five different concentrations were used for each xenobiotic, dissolved in DMSO present at a final concentration of 0.5%. The maximum concentrations used were 1 mM chloroquine, 0.5 mM dazomet, 2 mM imidacloprid, 0.5 mM thiabendazole and 0.5 mM 5-hydroxythiabendazole. Control cultures contained 0.5% DMSO only. Each well culture was replicated at least three times and dsRNA targeting *gfp* was used as a further control [71]. Predicted binding motifs were identified in promoter regions using TFSEARCH [72]. Promoter regions were taken directly from the *C. elegans* Promoterome and the number of SKN-1 binding motifs above a threshold score of 85.0 was counted. BLAST searches with protein sequences for *C. elegans* NHRs were carried out at NCBI (http://blast.ncbi.nlm.nih.gov/Blast.cgi) against protein sequences for *C. briggsae* and *C. remanei*.

## Supporting Information

Figure S1 **Workflow diagram showing the experimental scheme followed.**
(DOC)

Table S1 **Relative gene expression for transcripts significantly up-regulated (p≤0.01) after 1** **hr and 48 hr xenobiotic exposure as determined by qPCR analysis.** Genes analysed were those involved in cellular metabolism which had been upregulated ≥5-fold in preliminary microarray analyses. Gene inductions confirmed by GFP reporter strains appear in bold. Genes induced by more than one chemical are also indicated: ^A^ chloroquine and imidacloprid exposure; ^B^ dazomet and thiabendazole exposure; ^C^ imidacloprid and thiabendazole exposure
(DOC)

Table S2 **Putative homologues identified in** ***C. briggsae*** **and** ***C. remanei*** **protein databases from BLAST searching with predicted protein sequences for** ***C. elegans*** **NHRs that regulate xenobiotic responses.** The E-value is provided for each homologue.
(DOC)

Text S1 **Primer sequences used for qPCR analyses.**
(DOC)

Text S2 **Promoter sequences cloned into the Multisite Destination vector PDEST-DD04 for transformation of** ***C. elegans*** **.**
(DOC)
