# Supplementary figures and images for: Adaptive and Specialised Transcriptional Responses to Xenobiotic Stress in Caenorhabditis elegans Are Regulated by Nuclear Hormone Receptors
Source: PLoS One. 2013 Jul 26;8(7):e69956. doi: 10.1371/journal.pone.0069956 (PMC3724934; doi:10.1371/journal.pone.0069956)

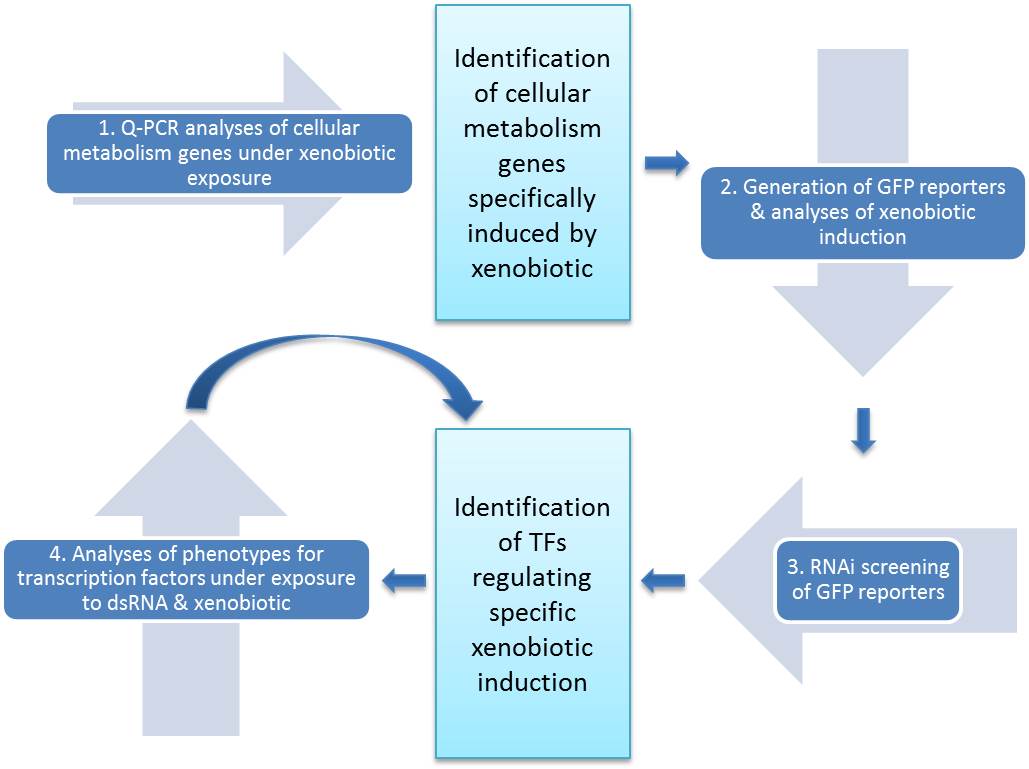

Supplement: Figure S1 — Workflow diagram showing the experimental scheme followed. (DOC) [file pone.0069956.s001.doc]
