# Supplementary material for: Adaptive and Specialised Transcriptional Responses to Xenobiotic Stress in Caenorhabditis elegans Are Regulated by Nuclear Hormone Receptors
Source: PLoS One. 2013 Jul 26;8(7):e69956. doi: 10.1371/journal.pone.0069956 (PMC3724934; doi:10.1371/journal.pone.0069956)
Supplement: Table S1 — Relative gene expression for transcripts significantly up-regulated (p≤0.01) after 1 hr and 48 hr xenobiotic exposure as determined by qPCR analysis. Genes analysed were those involved in cellular metabolism which had been upregulated ≥5-fold in preliminary microarray analyses. Gene inductions confirmed by GFP reporter strains appear in bold. Genes induced by more than one chemical are also indicated: A chloroquine and imidacloprid exposure; B dazomet and thiabendazole exposure; C imidacloprid and thiabendazole exposure (DOC) [file pone.0069956.s002.doc]

**Table S1a**

| **Gene family** | **DMSO control** | | **Chloroquine** | | **P-value** | |
| --- | --- | --- | --- | --- | --- | --- |
|  | 1 hr | 48 hr | 1 hr; 0.25mM | 48 hr; 1.0mM | 1 hr; 0.25mM | 48 hr; 1.0mM |
| **Cytochrome P450** |  | |  | |  |  |
| ***cyp -35b1*** | **1 ± 0.06** | **1 ± 0.23** | **3.82 ± 1.23** | **8.60 ± 0.67** | **0.035** | **0.006** |
| *cyp -35b2 ^A^* | 1 ± 0.02 | 1 ± 0.28 | 6.67 ± 2.01 | 632 ± 17.4 | 0.010 | 0.001 |
| ***cyp -35b3*** | **1 ± 0.02** | **1 ± 0.32** | **5.32 ± 1.55** | **1870 ± 125** | **0.001** | **0.001** |

Table S1b

| **Gene family** | **DMSO control** | | **Dazomet** | | **P-value** | |
| --- | --- | --- | --- | --- | --- | --- |
|  | 1 hr | 48 hr | 1 hr; 0.25mM | 48hr; 0.5mM | 1 hr; 0.25mM | 48hr; 0.5mM |
| **Cytochrome P450** |  | |  | |  |  |
| *cyp-13a10* | 1 ± 0.03 | 1 ± 0.03 | 1.31 ± 0.08 | 4.36 ± 0.03 | 0.048 | 0.001 |
| **UDP-glucuronyl-transferase** |  | |  | |  |  |
| ***ugt-13 ^B^*** | **1 ± 0.01** | **1 ± 0.03** | **1.60 ± 0.10** | **3.60 ± 0.17** | **0.014** | **0.001** |
| *ugt-46* | 1 ± 0.09 | 1 ± 0.04 | 9.74 ± 0.48 | 7.80 ± 0.39 | 0.048 | 0.013 |
| **Glutathione transferase** |  |  |  |  |  |  |
| *gst-4* | 1 ± 0.07 | 1 ± 0.03 | 1.80 ± 0.20 | 8.48 ± 0.56 | 0.010 | 0.005 |
| *gst-5* | 1 ± 0.23 | 1 ± 0.02 | 2.36 ± 0.10 | 12.7 ± 1.10 | 0.025 | 0.007 |
| *gst-12* | 1 ± 0.10 | 1 ± 0.06 | 3.35 ± 0.44 | 47.4 ± 4.25 | 0.030 | 0.006 |
| *gst-13* | 1 ± 0.06 | 1 ± 0.02 | 1.68 ± 0.12 | 3.86 ± 0.02 | 0.004 | 0.013 |
| *gst-14* | 1 ± 0.06 | 1 ± 0.37 | 1.30 ± 0.01 | 69.9 ± 3.72 | 0.005 | 0.003 |
| *gst-16* | 1 ± 0.09 | 1 ± 0.02 | 1.56 ± 0.10 | 4.56 ± 0.20 | 0.016 | 0.001 |
| *gst-21* | 1 ± 0.04 | 1 ± 0.17 | 2.82 ± 0.12 | 6.51 ± 0.17 | 0.001 | 0.003 |
| *gst-25* | 1 ± 0.20 | 1 ± 0.22 | 1.80 ± 0.20 | 24.9 ± 1.85 | 0.013 | 0.001 |
| ***gst-30 **** | **1 ± 0.09** | **1 ± 0.08** | **3.97 ± 0.09** | **44.1 ± 5.59** | **0.017** | **0.014** |
| ***gst-31 **** | **1 ± 0.12** | **1 ± 0.05** | **1.75 ± 0.10** | **46.3 ± 4.24** | **0.017** | **0.007** |
| *gst-39* | 1 ± 0.09 | 1 ± 0.02 | 1.48 ± 0.07 | 4.44 ± 0.19 | 0.006 | 0.001 |
| **Alcohol dehydrogenase** |  |  |  |  |  |  |
| *dhs-8* | 1 ± 0.09 | 1 ± 0.03 | 2.02 ± 0.09 | 4.39 ± 0.19 | 0.001 | 0.001 |

Table S1c

| **Gene family** | **DMSO control** | | **Imidacloprid** | | **P-value** | |
| --- | --- | --- | --- | --- | --- | --- |
|  | 1 hr | 48 hr | 1 hr; 0.5mM | 48 hr; 2.0mM | 1 hr; 0.5mM | 48 hr; 2.0mM |
| **Cytochrome P450** |  | |  |  |  |  |
| *cyp -29a2* | 1 ± 0.12 | 1 ± 0.04 | 1.98 ± 0.16 | 2.21 ± 0.09 | 0.016 | 0.001 |
| *cyp -33c1* | 1 ± 0.01 | 1 ± 0.16 | 1.81 ± 0.02 | 2.29 ± 0.04 | 0.333 | 0.009 |
| ***cyp -34a7*** | **1 ± 0.04** | **1 ± 0.16** | **1.40 ± 0.09** | **21.4 ± 1.22** | **0.013** | **0.002** |
| *cyp -34a9* | 1 ± 0.16 | 1 ± 0.04 | 2.39 ± 0.20 | 1.86 ± 0.08 | 0.015 | 0.001 |
| *cyp -34a10* | 1 ± 0.09 | 1 ± 0.16 | 1.09 ± 0.02 | 21.9 ± 1.61 | 0.663 | 0.004 |
| *cyp -35a3* *^C^* | 1 ± 0.05 | 1 ± 0.18 | 2.08 ± 0.16 | 5.26 ± 0.26 | 0.001 | 0.001 |
| ***cyp -35a4*** | **1 ± 0.18** | **1 ± 0.17** | **1.55 ± 0.14** | **2.88 ± 0.15** | **0.130** | **0.014** |
| ***cyp -35a5 ^C^*** | **1 ± 0.06** | **1 ± 0.04** | **2.14 ± 0.09** | **1.93 ± 0.08** | **0.010** | **0.002** |
| *cyp -35b2 ^A^* | 1 ± 0.07 | 1 ± 0.06 | 1.01 ± 0.08 | 18.9 ± 0.85 | 0.926 | 0.001 |
| ***ugt-25*** | **1 ± 0.08** | **1 ± 0.03** | **2.63 ± 0.21** | **5.30 ± 0.30** | **0.030** | **0.006** |
| *ugt-33* | 1 ± 0.06 | 1 ± 0.04 | 1.14 ± 0.12 | 3.99 ± 0.25 | 0.263 | 0.014 |
| *ugt-36* | 1 ± 0.14 | 1 ± 0.22 | 0.90 ± 0.15 | 3.60 ± 0.16 | 0.641 | 0.013 |
| ***ugt-37*** | **1 ± 0.08** | **1 ± 0.04** | **1.47 ± 0.18** | **3.17 ± 0.16** | **0.101** | **0.001** |

**Table S1d**

| **Gene family** | **DMSO control** | | **Thiabendazole** | | **P-value** | |
| --- | --- | --- | --- | --- | --- | --- |
|  | 1 hr | 48 hr | 1 hr; 0.125mM | 48 hr; 0.25mM | 1 hr; 0.125mM | 48 hr; 0.25mM |
| **Cytochrome P450** |  | |  | |  |  |
| *cyp-35a3 ^C^* | 1 ± 0.11 | 1 ± 0.14 | 10.9 ± 0.38 | 4.46 ± 0.25 | 0.001 | 0.011 |
| ***cyp-35a5 ^C^*** | **1 ± 0.05** | **1 ± 0.06** | **40.7 ± 1.57** | **8.35 ± 1.03** | **0.001** | **0.015** |
| *cyp-35c1* | 1 ± 0.02 | 1 ± 0.03 | 13.6 ± 1.23 | 1.35 ± 0.20 | 0.009 | 0.155 |
| ***cyp-35d1*** | **1 ± 0.10** | **1 ± 0.13** | **257 ± 81.2** | **79.0 ± 0.19** | **0.001** | **0.003** |
| **UDP-glucuronyl-transferase** |  | |  | |  |  |
| ***ugt-8*** | **1 ± 0.02** | **1 ± 0.01** | **24.6 ± 0.18** | **10.1 ± 0.05** | **0.001** | **0.001** |
| ***ugt-13 ^B^*** | **1 ± 0.01** | **1 ± 0.02** | **3.52 ± 0.01** | **2.63 ± 0.16** | **0.001** | **0.001** |
