# Supplementary material for: Adaptive and Specialised Transcriptional Responses to Xenobiotic Stress in Caenorhabditis elegans Are Regulated by Nuclear Hormone Receptors
Source: PLoS One. 2013 Jul 26;8(7):e69956. doi: 10.1371/journal.pone.0069956 (PMC3724934; doi:10.1371/journal.pone.0069956)
Supplement: Table S2 — Putative homologues identified in C. briggsae and C. remanei protein databases from BLAST searching with predicted protein sequences for C. elegans NHRs that regulate xenobiotic responses. The E-value is provided for each homologue. (DOC) [file pone.0069956.s003.doc]

**Table S2.**

| ***C. elegans*** | ***C. briggsae*** | ***C. remanei*** |
| --- | --- | --- |
| NHR-62 | CBR_NHR-62 E = 0 | CRE_NHR-62, E = 0 |
| NHR -120 | CBR_NHR-120 E = 0 | CRE_NHR-120 E = 0 |
| NHR -176 | CBG22840 E = 3 x 10^-47^ | CRE_09122 E = 10^-86^ |
| NHR -11 | CBR_NHR-11 E = 0 | CRE_14061 E = 0 |
| NHR -104 | CBR_NHR-104 E = 7 x 10^-171^ | CRE_20002 E = 9 x 10^-177^ |
| NHR -149 | - | CRE_27242 E = 10^-99^ |
| NHR -170 | CBR_NHR-170 E = 0 | CRE_NHR-70 E = 0 |
| NHR -175 | CBR_NHR-175 E = 2 x10^-151^ | CRE_NHR-175 E = 2 x 10^-160^ |
| NHR -180 | CBG23582 E = 10^-174^ | CRE_27865 E = 2 x 10^-170^ |
| NHR -199 | - | - |
| NHR -218 | CBR_NHR-218 E = 10^-133^ | - |
| NHR -268 | - | - |
