# Supplementary material for: Adaptive and Specialised Transcriptional Responses to Xenobiotic Stress in Caenorhabditis elegans Are Regulated by Nuclear Hormone Receptors
Source: PLoS One. 2013 Jul 26;8(7):e69956. doi: 10.1371/journal.pone.0069956 (PMC3724934; doi:10.1371/journal.pone.0069956)
Supplement: Text S1 — Primer sequences used for qPCR analyses. (DOC) [file pone.0069956.s004.doc]

**Text S1**

ama-1: Forward: TCG AAC GAC ATC CAA CGA TA

Reverse: TGC CAA ATG TCG GTA ATT GA

ahr-1: Forward: TCT CCA ATC AAC AGC TGC AC

Reverse: AGT TGG AAC AGG AAC GGA TG

cyp-13a10: Forward: GCA AGA GCA GAT GAT GTG GA

Reverse: CAG TTG TAT CGA AGC CAG CA

cyp-29a2: Forward: CAC CAG TTC CTG CAC TTC AA

Reverse: TGC TGT GAA GAA CCA TTG GA

cyp-33c1: Forward: AAG CGA GGG TGA TTC TGA AA

Reverse: TGC AAA ACG TAG CTG ATT GC

cyp-34a7: Forward: TCA GGA GAC CAC ATC GAC AA

Reverse: TAA GGG AGA CTC CAC GTG CT

cyp-34a9: Forward: CGA GAC TCT TGC CGT AGA CC

Reverse: TTC CAC CGG TAA CCT CTG TC

cyp-34a10: Forward: ACA GCG GTG CAC CTT CTA CT

Reverse: CAC CAC ATT TGG ATG GTT CA

cyp-35a3: Forward: TTC CAT TCG GAG TTG GAA AG

Reverse: GTT TTC CAT GTG GCT CGA AT

cyp-35a4: Forward: CTG CTG CAG AAG CAA TCA AA

Reverse: TTT CAA CAA GAA CGC GTC AG

cyp-35a5: Forward: CGA TTT TCA AAA ACC CGA GA

Reverse: CCA GGC AAT TTC TCT TTC CA

cyp-35b1: Forward: CAA AGA TGG AGC AGG AGA GG

Reverse: ATT GAA TCC TGC GAC CAA AG

cyp-35b2: Forward: GAG AAT CCG CAT GAT TTC GT

Reverse: CCA GCT AGC CAC AGA TCT CC

cyp-35b3: Forward: TCC CCA GGA TTT TGT TGA AG

Reverse: ATC CTG TCC AGC TAG CCA AA

cyp-35c1: Forward: CGT TTG GAA TTG GAA AAA GG

Reverse: AAC GGT TTC TCG AGT GGA TG

cyp-35d1: Forward: GGG TAC ACG TCT TCG TTG GT

Reverse: GCA GAT TGC AAA ATC GGA AC

dhs-8: Forward: GGA GTC TTG CTG GGA TAA CG

Reverse: TCG ATC ATC AAT TTG CTC CA

gst-4: Forward: TGG GCT GAT ATT GTG ATT GC

Reverse: TCC TTT CTT GTT GCC ACG TA

gst-5: Forward: CTT GGC TAT TGC CCA ACA TT

Reverse: GGG TCT CGA TCC ATT TCT TG

gst-12: Forward: CCG GAA AAT CAG TGG AAG AA

Reverse: GAA AAG ATC CGC AAA GGT GA

gst-13: Forward: TTT GGA TTC GCA GGA AAA AC

Reverse: TCA CCG TCC AGA TCA GTC AA

gst-14: Forward: TCT TCT CCC AGG TGT CAT CC

Reverse: GTT CGT GGT CTA GCC GAG TC

gst-16: Forward: AAC GAC GCA GTG GAA AAT CT

Reverse: CCG AAT TGC TCT TTT CCA GA

gst-21: Forward: AAT GCT GAC GCA TGA AGA TG

Reverse: GCC TTG ACG CAA TGT ATC CT,

gst-25: Forward: GAA CAT CTC GCA CGT CTT GA

Reverse: CGA AGA GCA CGT AAT CCA CA

gst-30: Forward: TGC TGC CAG TCA ATC ACT TC

Reverse: TGG ACG AGT CTC AAC CCA TT

gst-31: Forward: GGT TTC CTT GTT GGT GAT GG

Reverse: TGG AAT CGC GTA GAC CTT CT

gst-39: Forward: AGA AGT CGC CAT TCC AGC TA

Reverse: GCG AAA GTG AGA CCA TCT CC

hif-1: Forward: TGT GGA ATG CGT CGA TTT TA

Reverse: AAA AGC CAT TGC AAA ACG TC

mdt-15: Forward: ACA GTT CCC ACC ACT TCG AC

Reverse: GAA AGA CCG GGT CGT GAT AA

nhr-8: Forward: AAT CCG AAA AGC ATT GAT GG

Reverse: CTC CCC AAA TCC ACT TTT CA

skn-1: Forward: GGA CGT CAA CAG CAG ACT CA

Reverse: GAG AGC ACG TTG ATG ACG AA

ugt-8: Forward: AGC TGG CTG ATG GAG AAA AA

Reverse: CTT GGA TCA ATT GGC TGG T

ugt-13: Forward: CGAGATGAAACGAACTGCAAC

Reverse: GAGCCCTGTGATCTCGAAAG

ugt-25: Forward: ATG ACT CAC GGA GGT CTT GG

Reverse: TGC AAG CAT ATT CGC ATT TC

ugt-33: Forward: AAA TGG GTT CCA CAA ACT GC

Reverse: AAC TGC TGG TTT TCC CAT GT

ugt-36: Forward: GCA AGT TTC CCG ATG TGA CT

Reverse: TGC ACT TAG ACG TGG GTC AG

ugt-37: Forward: TGG CAG CCA TCT TGA TAT TTT

Reverse: TCT TGA CTT TGC GTG AAT CG

ugt-46: Forward: TGC AAG TGA AAT GAG CAA GG

Reverse: GTC GGG GAA CTT CTT CAC AA

Y35g12.2: Forward: ACT GCG TTC ATC CGT GCC GC

Reverse: TGC GGT CCT CGA GCT CCT TC
