# Supplementary material for: Adaptive and Specialised Transcriptional Responses to Xenobiotic Stress in Caenorhabditis elegans Are Regulated by Nuclear Hormone Receptors
Source: PLoS One. 2013 Jul 26;8(7):e69956. doi: 10.1371/journal.pone.0069956 (PMC3724934; doi:10.1371/journal.pone.0069956)
Supplement: Text S2 — Promoter sequences cloned into the Multisite Destination vector PDEST-DD04 for transformation of C. elegans . (DOC) [file pone.0069956.s005.doc]

**Text S2.**

Promoter sequences cloned into the Multisite Destination vector PDEST-DD04 for transformation of *C. elegans*

**cyp-34a7 (1053 bp)**

AATCTGAATAAATCTTTTATTTTGTGCATGTGATAAATTATATTGTTAAAAATATATGAAGGTTTTCGGTCGAAATTGAAAATAGGAAAAATAAAAATACTTATTTTTTCAATTATCAGGCAACCGTAATTCAATAAACTGGGGTTTTGTCGATCAACATCAACCAAATCACATGTGATTTCTGATCAAGTTTAAAAAATAAAACAGATATTTTTTGAAGAAATTATACATAGAATTGAATGTGAAAAAATCTGATTAGATGGAGTCAATATGTTCGGCCGAGAATAGTCAAAATTGCATTAGTAAAACATTCAAAATAAATTTGAAATACCGTGTTTAGAAAACATAATGTGCAAACTAAAAGAGTAATTAAAGAAGGGTAGTTGGATGTGGGGAATTTCCTAAAAAATTGTAGTGCATATTTAAAAACTTTCTATTTTATCGAAAGACAAGTCTTTACAGTGTAAAAAAATTAGCTAGTATGCTACAAATTTTGGACAACCTCGCAAATTTTGGCAAATTTTGGCAGTTGAAGCAAATAATTTTTAAGCTAAAATAGAAAACATAAATATTAAATTTTGACATTTTTCAAAATTTTGAAAGCCCGTAAAATGTTTTCAAATGTAGTGTGAAGTTGGCTTTTTTTGGAGTAACTGTAATTTATAAAATAATACAAGCCAATCTAATTTACCATGTTCTCTCATTAATTTGAGTTATTACATAATTCAGTTTTTGTTGAAGGTATGCCTAGGCGCTTTTAGGACAAATAATTAAAGTTTTTAGAGAGTCATACTTATTATAAATTTACAAAATTACAGTCAATCAAAAGCAGAAACTTTCAAAGAAAACACCAAATAACACAACACAGCCAAAGTGTGGTCTGTACCCTCAACTTAGAGAAAGCATACTTTTTCTGCTGCTGCTGTCTTTTTGATTTTTTCTCTATGATGCGTCTCATTCTCCTCCTACCGATTAGTTAAAAATGTAGTTTCTGTGAGATGGTTCAGATATATAAGTTGGCGAACAACTCTAGTTTAGTTGCATCTAGAAACAATG

**cyp-35a5 (583 bp)**

GAAATTCGATTTGTAAAACGTTCTTAATAGTCTTCGACTGTTTTCCAATTGTAGTTTAGATAAGAAAAAAACCAATAAATAGTTCAGTTTTGGACAAAAATGGCCCTATGTAAGTGCTCCAAGCATGTAGTATCTGAGACATTCTATCGAAACCGAAAGGTAACTTTGCATGCTGAATTTTACCAGAAAAACATTTTTCTAATGAAAAAATCTGGGAAAACCCAGTATCTGAATTTGTGGATTTCATAAAGAACACCTCGACATCTCACGAGCCAATTTATGAATATTTCTTTTCATAATTCTAGCAGACCCAGGTTATGCCCACTTTTTTGATATAATGTTTCAACATTTATTTTAAAATCTTCATAACTATTTTTGAAAATACGAAAAATAAACTTCACAAAAATCGTTGTCAACACAAACCACAAAATTTCTTTCTCTAATTCTCTTTTTTTCAATGTAATTTTCATTTTCTGTTCCCTGTTGTGTCACTTTAACTTTGAACAAATAAAATGAAGAGTGATTAGAAGAAAAACATATAAGAGTGGATAGAAAACGATTTTTGTCAGTCAATTAAATCAAAATG

**cyp-35b1 (703 bp)**

AAAGGAATTCACAGCTCAAAAAATAATAACTACCGGTTCAGAGATTTTAGTAAATAAATTTGTTTAATGGTTGCTTTGTATTGCAGATTTTGTTGTTCTCTACTAAAAAAACTCTCTTTTCTCATTTTTTATTTGTTTTTACAACACAGCTGATAAATTTTTTGGAGCTCCTCATTTCACGTCAATTCAGGCATGTTCAATGTTTTTATTATTATTTTAAACATATAGGTTTTTCGCATTTTAAAAAATGGGGTTTGTCGGTGGGATTTGTTTTAAATACATTTTCAATACATTCCAAAATTTTTCAGAGTTTTCCAAATTTTCAGTCAGAGTTTTTGGTAAATTATTAAATTTTCGGTAATGTGATTTTTTCGTGAAGTGTTGAGTATTGAGCAATTTGTTGATATTTTAAAAATAATGTTACACATAAAAATTTTCTGAATTGAAAAACGGAAGAAATTATAAAAAAAAACCAATTTGGCTAATTTTTATGATCAAATTTCAATTTACTCCCCTCAGAAATCAATAAACAGGTTTTGAAGTAACCTTCGATTTTCCCTTATCTTTATGACTAAACTGTATGTGGGACAGAAAACAGTAAAACAAATTGAAAACAATTTTCTCGCCAAAGCGTAGTTTGTATTCAAAGTTTCCTACAAATACCCACTGGAAAATTGAATTCTTAGTTTGATTTCGTTCAGCCATG

**cyp-35b3 (897 bp)**

AATCTTTAATGATAATTTATGGGATCTGTATTTCTCTTTCTGTCAATAAAAATTGAAAATGATTTTTACATTCTCAATATTTTCTAAATCATGTTTCGTGAAGCTGAAGAGTAAAATTCGACATTTAGAAGGTTTCGTTAGAAAAATGAAAAGTGTAGTGCCAGAGGGGACTTTATCTAAAACAGGCCTGAAGGTTCGACCCGCGTTACAGTTCCAGTCTAAAGTAATAACACTAATTCAAAATAATATATACGAAAAAAAAACACTTGAATATTATTTGATTTTTAAAGATTTTCAATTTTGAAATTATCAAATTTCCTTGAATTTGGGAATTTTTGAAGAAGTTTCAGATGCAGGTTTGAAATCCTAGAATGTGCAAGTATGAAAACTGAAACAAAATGTATTTATACGACTTTTTTGGTCACTGCCAAACTTATAATCGGTCAAAACTATGTTTGCACAAATTTCTAACATTAAAAATAAACGATTTTAATTTTTTTTTGAAAATTATGCCTGTATACATTTCAGCATTATAAGAGCGTTTTTAAGCGATTCCCTACTGATGATACTGTAGCATTCTAAAATTATTGTAGCTTAATAGCTATCTAATTTGTAAAATTAAATTTAAAAAAATAAATTTGAAGTGGATCTATTAGAACCTTCATACAATATTTCCTACTCTTTTAAATTTGAAATTTTTCGAGTCAGTGCTAGTGATAGATAGAATACATCCATTCCGTAGTTATCTACGCTTTCCTCTTGGAATCAACACATCAAAACTCAAAGTACGCCTTTATTAAAGAACCGTGCTTTGTAGTTTTAAATTACTTGCTTCCATTGTTTGTAGCCTTTCCTTATAAAAGATAGCAGGTTCTGTTTAACTATCTCAATTTCAAAATG

**cyp-35d1 (493 bp)**

ATTGATGTAGTAAGATTGCAAAAAAGGAACTCAGAACAGTAAGAAAGCCAAAGTTAAAAGCATTGTTGTCCTGAAAAATCCTTATTAGTGTGTGATAAAAATAAATTTCACAAGTTGGACAGTTATTATTTCACAAAATAAAATATTATTTTGTTGTGTGTACTTTACAATTGACGAAAAGATCAAACCGACGCAAAAATGATCAATATAATCCGTTCATATTTGTTTGGTAAAGCATTTTTCTGCTAATCAAAAACTGTTGGTGCAAAATAATCGCACGTTTTTTCGTTTTTTTTTTAATTTTTTGGTCTCAAAATTACATAAATTTTCGGAAACATTTCTAACGCTGAAAAAAACATTTAATTGTGTGAAGTGTAGCCGTGAAAATGTGTTAGGTGTTGCTACCCTCTTATCTTCAATCTTATCATGTTTTTGTCTCCTTTATAAAGAATTGCCGGTGAACTTGAAGTTCAGATGTATAACTGTTTCTATCATG

**ugt-8 (991 bp)**

AAGTTTTTAAAAAAGAAAGAGAATTTGTTTAAAAGTACCAATAACTCCTAATTTCAAATTTCCGAATCTCCGCTCTCAGGTTCCAGAGATCAATAAAGTCGGACTAATTGCTCATTACTATCTTGATATCGTTGCATTCCTTCTACTTTCTGCTATTTTCATGATTTATGTTTTTATCAAAATTATTTATCGATTAAAACTGAAACTTGTTTCGACTAAACCGAAAGAGGAGTAGCATATTTTAAATAAATATTTTGCATTATATTTAATTTACGTACGTACACATTCAGTGTGTTTTTTTTTTCAGAAAATATTTAATAAACTTGCGCAACAATTTTGAGAGTAACAGCTCTGAAAAATTATTCGTTTTTAATATAAATATTGCGTGTAATTTGCATGCTACTTTCAGTTTTGCGAATAAGCCTCTTTTTCATATAAATTTTTTTATTTTGAAATGCAAAAGTTCATGTGATCAAAAATTTTAATTTGAATTTCAAAACAGACTTTACAATGTGTACCGAAGTCGAACCGAGTAATTTGGGAAAATAAAACATGCCCAGAACTTGTTTGCTAATTGAGAAAAAACAACAAAAATTAGTTCCTTAATTTAATTTGAATTTAAGGATATTTTTAAAAAATTCGCAGACAACTTTTTTTAAATAAAAACTGAAAAAATATAAATCTAATTTAAAATTTCAACCTAACAAATTAAGAAAGTTTTTGATCCTAGTTATACTATACCATCTTCCGAACAAAAAACACAGAATTTGAACAAATAGCCTCATCACGTCAAACCCTATTATTATTCTCTTCAGTGACATAATAATCTCTAAAAAGTTTCCCCGTGTTCAGTGTAGTATCACTCTACACTCAACACGAAAAACCTATAAACACTACAAACTTCATATGGACACGAAACACGATGGTCATTCGATTAGTGACTGTATAAAAGAAAAGTGACTATCACTGTCAGTTGAGTATCTAGTTGAACATG

**ugt-13 (630 bp)**

AAATTATTATGTTCCTATTTCTTTTATCAAATAAATGCAGTTTTAAAATTTTGGACTTTTCTGAGAACGTACAGCAATAAATAAAAATCTAAAACCAATCACATTCAAAAGGTCGGAGCAAGTTCGGAGCTCCGGGATTCAAGGTCACAATAATGAAATTGTTTTTTTATTGCTTGACATTGATCGAAATTAATTTGTTATTTTTTGCAAAATCGAAAATGAATATTTTTGAATTAGAAATGTTTTTACAAAATTTTGAACCGCCATAAAAAATGTTGAAAAGTTAAAGTTTTATTACGAAATTCGTACATTTGAAAACCTTTTGGGTCTACATGTTCAAAATCGCCCGAACCGTTAGTCTTCCTTTAAAGTCAGTTATGACTGTGTTCTGTGTCTCCTCGACTCTGTTTTCTGAATTGTCATCACACCAAAAGACCAATCTTTAGATCTTTGTATTTCTTTTCATTACTTGCTATCAAATTAGCCATGAAAAACATATGTCATCATATTACTCACTCAAAATACTACAAACTACACTGACGAGGTTACCGTTTGATCTTATCATCTCTTAAATTAGTCGGGGTATATAAGAAGAACAAATCGAGTACATTGTTTCAAGAAAAATTCCCAATG

**ugt-25 (1327 bp)**

AGAACTGGTATTGTTTGCATTTCATTTGATTGTTCAATTTACGGATCTCTAAATAAATTCCTCTGATTTTCAAGTTGTTCAATTAATCAAAAATTATGCTCAAACTTTTAGTTTTAATTTTCTAAAAAATTGAATTAAAAAGGCAAATAAGTGCCTTATACCTAAAATGCTCTCTAGCACTATCAGAAATATTGATTGCAGTTTAAGACATTTTTCAAGGTTTGTGATCTATTTTTTATGTTTAGATTACTGCAGAGAACGAAAAATCTAAATATATAGCACAAGAAAACACACCCACAATGAACAAACCACACTTTCAATGCGTGATCTTCATGATCTTCATTGCAAGTGACAGTTTTTCAATGAACGCGAATACTTTGTCTGTGCCTTGTCTATAGATCAAGTTACCACTCACAAAGAATAACTTCCTATTAGGGAAATAAATACAGTAGAAGTTTACAAGTTCGCACCCAGAGATCCTAATGAGAAACTAGCGACAAAAATCATTTGATTGTTTTCAGACGAAGACATCGAATTTTACAACCTAATTAACCGGAATTGACGACTTGGAAAACTGGAATGATCTATGTACCAAGTATATGTACCAAGTTCCTACACAATTACCCAATTAGTGAATAATAGTGCAGTTCTCAAATGTACAATATTAGTGTTTTACATTTTTAACCTAGAACATGAAAAATCTTGGCCAGTTAGGGCTACAAATTGGATTAAAGAAAACGTTCTGTGTCCGGTTTTTCCGGATTCTGGACGATCCGGCGCCTGTTTGTTTTCGGGGTTACAACATGTTTTTCAAATAAATTTTGTCTTTGTTGCAGTGTTCATCTCGAAAAGAAATACCAATTGAAATGCAAAAAATTTCATGAAAAAAAATTAATTTTAAAAAAAATAGACAACTCGGCAAAAGCAATGTGAAAACTTGGTTCCATTATTAAAAAGCGTGCGGAATACTTGCACTCTGCTAAAATTAAAAACTTTGGGTGCAATGAACAAAATTCAACTTTGTGGGTTTTTGAACAAAAATCTGTCCGAGAAAAGCTATGCAGAGATATAAATATCCAATCACATGAATTTCATGTTCACTAGAACACATTAATAAAGCAAATAAACTAGAAAGTATGAATGATTCGATTTCACAACATTTGGTCATATCAACATCATCATCCATATCAGTACAGTGTCTTTTCTCAAATACGAAGACTACGGAAACTACATTATTTCAGAACTAATGGGCGGAGAGGAACTCCTTAACTAAAGAACTTATAAAAGGAAAATGAAAAAGTGACAACAGCAGTCACATTAACACATAATG

**ugt-37 (353 bp)**

GATGCATGGAGTGTTTGATAAAAGCGCTTTCTACCCATGTGTAGCCTCTTCAATTTTTGTTATAATGGTTTTTTTTTTGAATAATATAATTATTTATTTCAGTGGATTTTTAACTAAATTACTATCTACCAGGAAGTAATTTTTTTTCAAATAGTCCAACCAAATCGTTAAAAAACAGAAAAAAAAACAATAATATGAACTTGAAATAACAACAAAAGTCTAGCCTAGACATGGTCACACACACTTTATACATTAATGTGACCAATTTCTGACTATTTTGTGTTATCTGCCGATACAAATGAGAGACCTTATATAAATAGTGAAAAATAAATTGTCTGAAAACATTGATCAGGATG

**gst-25 (2000 bp)**

TACCGTGTGGGAGTCCTAGCTTCCATAACGCTCTCTATTAGAACTGAACTAGCTGAAAAAGGTAGCGGTTTTGAAAATGCGGAGCCCTAATGCATGCATAAACACACGAAGTTTGACCTAAAATGTTTGAGACGTGGTTCATCGACTTCTACAACTTACGTTTAATAACACTCCATGCTCCGTACTGAACCTTTCTCCAGCGAGCCTTGACCAGCCAATCGGTAACCTTCGAGATCAACTCCATCACTGTCTCTGGGTCTTGCTTCATCATCTGAAAACTTCAGAAACTTAGTGGCTTACTCCATATTAAGATGTCATACCTGATCAAACTCTGCAAACTTTCCAGCTGTGAAGAAAACCTTTGTATTTCCAAATTGAAAATCGTTCTGGTCGAGACCAAGCGCGTGGAACAAGCACTTGGAGAAGAGCCGGGGATCGAGGCGAGCCAGACTTGGCGGAAGATTCTTCTCATACATCGCGTAGAGATCTGCAAACGATGTTCTGCTCGGGAACCCTTCTTGCATCAAGCGGAGCACAGAAGCCATTCCAGCACATTGGAGTTGGCCGAGAATCGCGGAGCCGTCAAAGTGCCATGCTTTCATCTGACTGTTTGGCTTCACACAACGCACAAAGTGAGTTCCCTGGAAAAATTTAAAGATTTTTTTAATGGAAAGTTATTCGGGAGCTTACGGTATTGTTGAGTTTGTCCAAGAGAGAAGACAATTGAGACTTGAACTTGGCGCCGACGCTGACCGCCTTCAGACGTCCTCCGGTCTTCACTGCTCCGGTTGCTTCACTAGTGAACAGGGAGACAACGAGTGGGAAGCTGAAAAAACAAGATATTAATTTTTAGAAAGAATCAGTTATTATATAAGTTTTGCTCAATTTTATTTTATTAAGACACACCAAAATTTTCTCATTTTCCCCCTTTCTGATCCTTTTTTCAGATACCTTTTTCACATTATTATAGTAGTACTACTGTAGAAGTACTGTAGGAGTACTTTAGGATTACTGTACTTTTGAAGGGATATTGGTTTGGGGTTAGTGGAGGAATATGGTCGGGGCACTGTAGTAGTACTGTAGAGGTACTGTAGGAGTACTGTAGGAATACTGTAGTTTTGGAAAAATTTACTTTTCGTCTTTTGAAGGGATATTGGTTTGGGGTTAGTGGAGGGATATGGTCGGGGTACTGTAGTACATCTGTAGTACAACTGTAGGAGTACTGTAGGAATACTGTAGTTTTGGAAAAATTTACTTTTCGTCTTTTGAAGGGATATTGGTTTGGGGTTAGTGGAGGGATATGGTCGGGGTACTGTAGTACATCTGTAGTACAACTGTAGGAGTACTGTAGGAATACTGTAGTTTTGGAAAAATTTACTTTTCGTCTTTTGAAGGGATATTGGATTGGGTTTAGTGGAGGGATGTGGTCGGGGTGCTGTTGTACAACTGTAGAGGTACTGTAGGATTACTGTAAGATTACTGTAGTTTTAGAAAAATTGGCTTTTCGTCTTTTGAAAGGATATTGGTTTGAGGTTAGTGGAGGGATATGGTCGGGGTCCTGTAGTACATCTGTAGTACAACTGCAGGGGTACTGTAGGAGTACTGTATTTTTGGAAAAAATTACTTTTGGTCTTTTGAAGGGATATTGGATTGGGTTTAGTGGAGGGATGTGGTCGGGGTGCTGTTGTAGTACTGTAGGGGTACTGTAGGATTACCGGGCCAAAAATCGCAAATGAAGCTTGTGCACAATCCTTTGGTGCTTATCAATAGTTGCACCACCGCATTGAGGCACGACTTATGTCAAAAATCACATTTTAACCTTCAAATCTCCCGGGCGACCATGGAAACAAAATTTGCCCCTCTTTTGTGAATCTGATCATTCTGTGTCTTTTTGTCATCATATTTCACTGCGTCTCTTCACAAACATTATGTCGATGTTTGTATTGTCTACAGTTCACTGGGTATAAAATACAATCATTGTCTCTTAGTGACTTATCAAAATG

**gst-30 (1249 bp)**

ATGTCTTCAGCTCTGAATTCCAATTTTGATGTAAATTATGAAAATAACAACAAATCCAAGACAATTGCGAGATCTACAGTACTCCTTTAAATCTAGTTGGTCGTTTCGAGACCGGGTACCGTATTTTTTCGTAAATTGCCAGGTAATTAGTGAAGAACGGAAATTTAAATTAATTTTTTTTATTAATTAAAGTTTACATTGTAATAGTCATTTTGTTAGATATGAACTGTTCTCAATTCTTCATTATTACACTAGTGTCTCAAATATGCTGACTATAAAAAAGAGCATTCTCAAATCACGTAAGACCAACAGAATCACACCTTAATATTAGCAAGGATGATAAATGTGGCAAGACGCGTCTCTAGTTTCCTCGATTTTCTCCCAATTCCAAGCAATTGTCATCATTTTGGCAACCTTTCTCGTACATTTTTAATGCCGCCTTACTTCGAAAAAAAAACACGATGAGAATGACAAACAAATTGAATGAAGGAGACAGTATAAAAGAGAATGTTTGAATTGGTCTTTCATCATTCGACTCCAAACACACTATGGTCCACTATAAACTTTCATATTTTCCAATTCGATTTGCTGGAGAAATTCCACGACAGATTCTGGCATATGCCGGGCAAAAGTTTGAGGATCACCGGATTCCACAGGCTGATTGGCCAGCTCTCAAGTCGACTATGCCGTTCGGAACTCTTCCTGTCCTTTATCAGGATGGGAAACCACTTGGACAATCCCATGCAATTTCTCGTTTTCTTGCTCGCCAATTTGGAATAAATGGAAGATGTCCATGGGAAGAAGCTCAAGTGAATGCAATTGCAGATCAATTCAAAGATTATCTCAATGACATCCGATCTTATAATCTCGTGAAAATGGGATTTGCACAAGGAGATGCCGATGCTCTTTACAAGGACACATTCCTTCCAAATTTCAAGAAAAACTACCAATTCTTCACCAATTTCCTTAAAGCCAGTGGATCTGGATTCTTGATCGGTGACTCTGTCACCTGGGTTGATCTGTTGATTGCTCAACATACATCAGATCTTCTCAGTGACAGTGGATCTGTGTTTGCTGCCAGTCAATCACTTCTAGACGAATTCCCAGAACTCAAGGCTCATCAAAAGAAGATTCATTCTATTCCAAATATCAAAAAATGGGTTGAGACTCGTCCAGTTACACCATTT

**gst-31 (1041 bp)**

ATTAGTTGATTGGAAGAAGAGAGGAAGGTGTGAGTATTGGAGTAATAAATTCAGAATCGCAGAATAATAGTTTTTTAAGAGAAAAAGTTCACGTAAAATTAATTTATTTAATATTCAGAATATATTTTACTGACTAAATAAATGTACATTTGGAGTATAGTCTGCATCAGTGCAGGATCTGGGAGCTCATCCGTTTGATACAACTTGTGCGAGCCAGTCGGAACTTAGTCTTGCAGGCTCCATTTGGCTTGAACCTCAACAAGCCACTTAAAAATTCGTCTTGCATGCATGACTAATGGTGGAAATAAGATTTATTAATACCGTGATCTTTAAATTGAGCTAGATGAATTTCAAAATCCTAAACAGAAGAGTTTTCTGAATTCTTGAATTTTCTGGGATTTCTTTATTCTCTCTTTCTCACATCTGCCCAGTCTAAGCCTAAGCCTAAGCCTAAGCCTAGACCTAAGCCTAAGCCTAAACCTATGTCTACTCTCATGATATTTACTTTTCAAATTAACACGTGAATTTAAAAAATGAATCCCTCGGTTAAAATGGCTTCAAGTAAATTTTTACATGTTTTCCGTCACACCACAAACCACATTTTGTCACGAAAGTTTCTGGAAAATGCTGACAAACATCGAGTGAAAGGAAATTACTACTAGAGAATTAGACATCCTGTTAAATAGACCAAGTGGGATTAAACATCAAAGTATGAGACGCAGAGTGGAGGAGGAGGGTTTTTGGAGAGCGGTGTGTATTTTTGGAAGTTTTTCTTATAAGTTGAGTTTTTGAATTGACAAGTTTATAAAAAACCATTGTCATTTAACCAACTGAAATTTTAAATTTTAAATGTTTTTTTTAAATAAAACTGTAGCATCACAATTTTCTATATAACATTTTTCCGCTTTTTCTGAATGTTAATTGCGTCTAAAGTAACACTAACAATATTCCCAACCTTGGTTTTTTTCAAAAAAAAAAGTCTCTAATATCTCTTTTAAGCCTGAAAGTTAACATATAACTTATAGTTTCAGTGAAACCACTATG
